# The eyes know it: Toddlers' visual scanning of sad faces is predicted by their theory of mind skills

**DOI:** 10.1371/journal.pone.0208524

**Published:** 2018-12-06

**Authors:** Diane Poulin-Dubois, Paul D. Hastings, Sabrina S. Chiarella, Elena Geangu, Petra Hauf, Alexa Ruel, Aaron Johnson

**Affiliations:** 1 Department of Psychology, Concordia University, Montréal, Québec, Canada; 2 Department of Psychology, University of California Davis, Davis, California, United States of America; 3 Department of Psychology, University of York, York, United Kingdom; 4 Department of Psychology, St. Francis Xavier University, Antigonish, Nova Scotia, Canada; University of Pécs Medical School, HUNGARY

## Abstract

The current research explored toddlers’ gaze fixation during a scene showing a person expressing sadness after a ball is stolen from her. The relation between the duration of gaze fixation on different parts of the person’s sad face (e.g., eyes, mouth) and theory of mind skills was examined. Eye tracking data indicated that before the actor experienced the negative event, toddlers divided their fixation equally between the actor’s happy face and other distracting objects, but looked longer at the face after the ball was stolen and she expressed sadness. The strongest predictor of increased focus on the sad face versus other elements of the scene was toddlers’ ability to predict others’ emotional reactions when outcomes fulfilled (happiness) or failed to fulfill (sadness) desires, whereas toddlers’ visual perspective-taking skills predicted their more specific focusing on the actor’s eyes and, for boys only, mouth. Furthermore, gender differences emerged in toddlers’ fixation on parts of the scene. Taken together, these findings suggest that top-down processes are involved in the scanning of emotional facial expressions in toddlers.

## Introduction

Infants’ interest in and attunement to human faces has been documented for decades [1–2]. Accurate identification and understanding of emotional expressions is critical for the social-emotional development of children [3]. Specifically, infants not only differentiate but also categorize facial emotional expressions in the first year of life [4–6], and can accurately match emotion labels and emotional faces by three years [7]. However, in comparison to adults, little is known about how infants and children scan emotional faces and identify emotional facial expressions.

Within the adult literature, some research examining the bottom-up mechanisms of emotional face scanning found that adults have a general bias to look at the eyes of an emotional facial expression across tasks and settings [8]. However, most findings demonstrate that adults change their scanning patterns of faces, focusing on the facial features most informative of each emotion, in order to bring together critical perceptual information. That is, anger, fear, and sadness are more easily recognised by adults from the top half of the face while happiness and disgust are more easily recognised from the bottom half [9]. More specifically, adults appear to focus on the eyes when decoding a sad facial expression, while the mouth appears more informative for the recognition of happiness [10–12].

Contrary to the adult mechanisms, little is known about these same bottom-up processes in infancy and childhood. Although there is much research examining the scan patterns of infants and children when examining emotional faces, these studies examined scanning patterns by collapsing across various emotional facial expressions. Within this body of work, there are mixed findings concerning the scan patterns of children over the age of 4 months. While some authors demonstrate that young children have a bias to look at the eyes as adults do [13], others highlight the emergence of a preference to look at the mouth when scanning emotional faces, which continues to increase with age [14], but reverses in older children. By the age of 11 to 12 years, children look more at the eyes again, which continues as they approach adulthood [15]. Finally, other findings show an equal distribution in scanning the eyes and the mouth [16]. Interestingly, an important study by Hunnius and colleagues (2011) examined the visual scanning patterns of individual emotional facial expressions from infancy to adulthood. The authors found 4- and 7-month-old infants, as well as adults, spent the longest time looking at the eyes across all emotional faces (neutral, happy, sad, angry and fearful). No other study, to our knowledge, has examined how infants and children scan individual emotional expressions. Due to the conflicting findings regarding young children’s scanning patterns when examining individual emotional facial expressions, the first goal of the current study was to determine if very young children, like adults, focus on the top part of the face (i.e., the eyes) when exposed to sad faces.

In addition to the bottom-up mechanisms driving the scanning of emotional expressions involved in emotion identification, Teufel, Fletcher and Davis (2010) presented a novel perspective on the links between the basic perception of social stimuli and theory of mind (ToM) skills. They argued that the representation of social stimuli as mental states contributes, in a top-down fashion, to the fundamental sensory aspects of perceiving social stimuli. Teufel and colleagues (2010) proposed that the “top-down modulation by ToM, a cognitive process specific to the social domain, achieves two key goals: prioritization of the most important social stimuli and disambiguation of informationally noisy perceptual signals” (p. 379). Extending from neurophysiological studies linking ToM to cortical and subcortical areas supporting visual perspective-taking (VPT) and the understanding of others’ emotions, Teufel and colleagues (2010) argued that the frontotemporoparietal and mirror neuron systems play complementary roles in shaping social perception. An experimental manipulation of adults’ mental-state attributions regarding others’ capacity to see provided evidence for top-down modulation of the gaze-following response [17] and gaze-perception system [18], providing evidence that ToM skills influence social perception, in addition to the previously discussed bottom-up mechanisms.

Indirect support for this top-down processing hypothesis comes from studies examining face processing in individuals with ASD with deficits in social cognition, revealing that individuals with autism scan faces differently than typically developing individuals by gazing less at the eye region of the face [19–21]. However, the findings for looking at the mouth are mixed. Some findings demonstrate that individuals with ASD look more at the mouth [20–22], while others reveal that children with ASD between 3 and 5 years of age look less at the mouth [23]. Furthermore, the degree of social and communicative competence of adults and children with autism is predicted by fixation times on mouths and objects during social interactions, but not on eyes [21, 24]. In fact, a consistent finding in the literature reveals that individuals with ASD base their emotion judgments less on information from the eyes region and more on information from the mouth region [22]. Overall, although some findings examining emotional faces scanning in individuals with ASD find no difference in their scanning patterns when compared to typically developing individuals [22, 24], most findings suggest that individuals with ASD look less at critical areas of the face than do controls.

While the top-down argument is drawn primarily from research on adults, there is evidence for some forms of top down effects on emotion processing in children. A recent study in 7-month-old infants suggests that those from Western cultures rely more on the mouth to visually discriminate emotional facial expressions than do Eastern children [25]. A more direct effect could be found in the interrelation between emotion processing and social cognition, given that emotion understanding involves the perception of a relation between a person and his or her perceived environment as signaled by emotional expressions [26]. Among socio-cognitive skills, theory of mind is the attribution of mental states to oneself and others. In support of this contention, it has been demonstrated that patterns of emotional face scanning related to social-cognitive skills in 5-to-6 -year-old children with autism as well as typically developing children[27]. Because some forms of theory of mind emerge in late infancy or early toddlerhood, it is therefore plausible that very young children’s attention to emotions and their visual scanning patterns of emotional facial expressions are guided by their nascent social cognitive skills [28–29]. Together, as eye-tracking research has shown that the visual scan patterns of infants differ for different emotions displayed in faces [13, 30], and false belief attributions influence how toddlers visually anticipate another’s actions [31], toddlers’ ToM could also be expected to relate to their visual attention toward emotional facial expressions. Thus, the second goal of this study was to examine the associations between two aspects of toddlers’ early ToM (emotional and visual perspective-taking) and their visual attention to a dynamic facial expression embedded within a social context.

The acquisition of perspective-taking skills in toddlers is two-fold: it involves the development of understanding that others have emotional states (emotional perspective-taking, EPT) and that others perceive and have knowledge about the world (visual perspective-taking, VPT). It has been shown that 2-year-old children can attribute the correct emotion to a character who did (happy) or did not (sad) find a desired object (emotional perspective-taking)[32]. Toddlers can also comprehend that others may have different non-emotional experiences compared to their own, such as understanding when they can see an image or an object that another cannot, termed Level 1 knowledge (visual perspective-taking) [33]. Whether Level 1 knowledge or early emotional perspective-taking also contribute to how children attend to dynamic social situations involving emotion remains undetermined. In order to address this gap in the literature, the current study examined the potential distinctive relations between these two dimensions of early ToM and toddlers’ visual attention to a dynamic facial expression embedded within a social context. As in the original visual perspective-taking tasks by Flavell and colleagues (1981), we administered two level 1 visual perspective-taking tasks to answer this question, in addition to an emotional perspective-taking task adapted from Wellman and Woolley (1990). Further, we opted to embed the emotional facial expressions in a social context as it is now recognized that context is an inherent part of emotion-perception [34].

Finally, the child’s gender might also play a role in the relations between ToM and emotion processing. Studies of early theory of mind skills typically do not find gender differences, and when these are reported, they are usually weak and accounted for by girls’ more advanced verbal skills [35]. Nevertheless, past work examining gender differences in emotional facial expression scanning in adults has demonstrated that females look longer at the eyes than men do, when correctly identifying an emotional facial expression [36]. Additionally, young girls may display more advanced understanding of others’ emotions [37], and they also seem to be more attuned to others’ facial emotions [38–39]. Therefore, we expect that girls might scan emotional facial expressions differently than boys do, due to their greater emotional understanding.

Very young children’s attention to and perception of the affective information of emotional facial expressions is hypothesized to rely on the integration of bottom-up information provided by the stimulus, and top-down influences by context, channeled by ToM. These include the regulation of their visual attention for the correct interpretation of emotional cues and avoidance of distracting, visual stimuli in complex social scenes [18]. Thus, early emotional and visual ToM skills should modulate visual attention to a sad emotional expression, which might differ based on gender. We tested this proposal by assessing toddlers’ emotional and visual ToM and by using eye-tracking to examine their visual attention to a dynamic scene in which an actor displayed sadness after her toy was stolen. Toddlers with more advanced ToM (demonstrated through their perspective-taking skills) were expected to direct greater attention toward the emotionally-salient components of the scene–that is, the face, and particularly the eyes, of the sad actor–and less attention to other stimuli in the scene that did not carry emotional information.

## Method

### Participants

A total of 67 toddlers participated in this study. Sixteen children were eliminated from the analyses due to general fussiness (n = 1), incompletion of the ToM tasks (n = 1), and incompletion/fussiness during the eye-tracking procedure (n = 14). The final sample consisted of 51 children (20 females) whose mean ages was 32.02 months (SD = 2.77 months, range = 29–38 months).

### Materials and procedure

Ethics approval for this study was obtained from Concordia University and St Francis Xavier University Human Research Ethics committees. Children and their caregivers first spent a brief period of time in a reception room in order to familiarize themselves with the experimenters and the environment. After caregivers completed a consent form and a short demographic questionnaire, they were invited into the testing room with their child. Task order was counterbalanced across all children.

#### Eye tracking task

**Fig 1:**
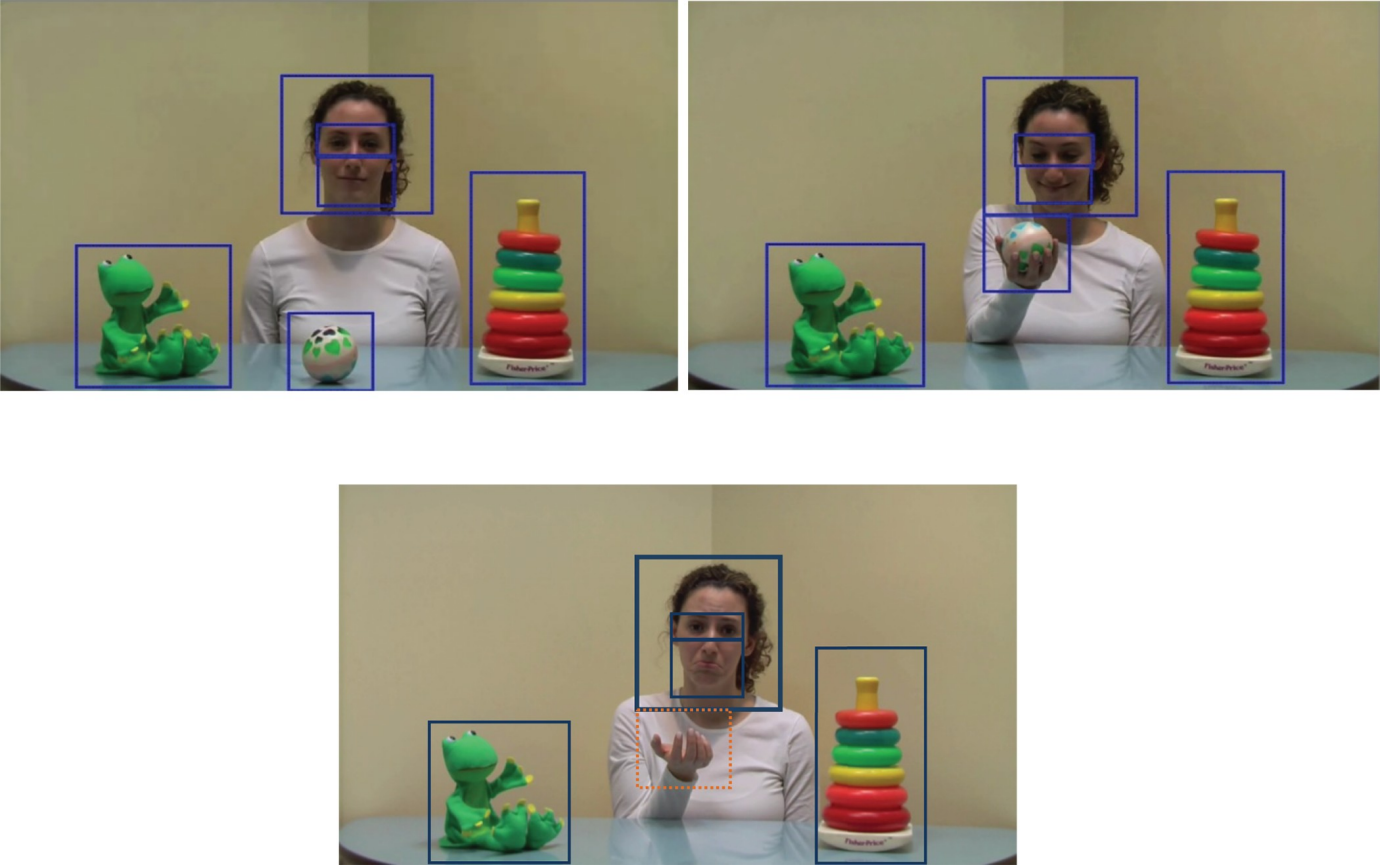
Video still frames of the pre-sadness (top frames) and sadness (bottom frame) segments with identified areas of interest.

Stimuli were presented and data collected using a PC running Experiment Builder (SR Research, Ottawa, Ontario). Participants viewed stimuli on a luminance calibrated video monitor (Viewsonic 19" CRT, 1024 x 768 pixel resolution, 100Hz refresh rate). Eye position was acquired non-invasively using a video-based eye movement monitor (EyeLink 1000/2K, SR Research, Ottawa, Ontario) in the remote monocular head-free mode. The EyeLink system recorded monocular eye position with a sampling resolution of 500Hz, and a spatial accuracy of 0.5 degrees of visual angle (manufacturers specifications). Participants wore a small target sticker above the eye to be tracked, allowing the eye tracker to compensate for head and body movements. After completing a 9-point gaze calibration (standard Eyelink locations, calibration average accuracy had to be < .5 degrees visual angle), an animated stimulus (a small dancing bunny, subtending approx. 2 degrees in visual angle) was presented to direct the child’s attention to the center of the screen. When the researcher judged that the child was fixating on the bunny, they initiated the experiment. Then, a 40 s video clip of a female actor and three toys (a frog, a ball, and a stack of rings) on a table was presented. The actor in the video has given written informed consent (as outlined in PLOS consent form) to publish her picture. After gazing at all three objects, she picked up the ball and smiled (12.5 seconds). At this point, the video showed a hand that entered the scene, quickly took the ball from her hand, and took it out of the scene (i.e., stole the ball) while the actor briefly looked surprised (2 seconds). The actor then expressed a sad facial expression, while gazing straight ahead (i.e., directly at the observer). After expressing sadness for 14.5 s, she actor sighed, looked at the other toys (5 seconds), picked up the rings and smiled (6 seconds). As shown in Fig 1, the following areas of interest (AOI) were analyzed: face, eyes, mouth, and distractors (frog, rings, ball/hand). In order to assess children’s visual gaze behavior during the video, the amount of time the child looked at the areas of interest (e.g., the face) was computed separately for the segment before the actor’s ball was stolen (pre-sadness segment, 12.5 s) (Fig 1, top frames), and for the segment in which the actor is displaying sadness (sadness segment, 14.5 s) (Fig 1, bottom frame). As this is the first study to examine young children’s scanning of a specific emotional facial expression (i.e., sadness) following a negative event, we focused on the pre-sadness and sadness segments. These times were then converted into proportions, due to the differences in duration of the pre-sadness and sadness phases of the video clip, in addition to differences in size of the AOIs. These proportions were calculated by dividing the amount of time the child spent looking at the actor’s face by the total time attending to the screen, for both the pre-sadness and sadness segments. Also, the difference in looking time was computed by subtracting the amount of looking time to each area of interest (e.g., the face) during the pre-sadness video segment from the amount of looking time to same area of interest during the sadness segment.

#### Emotion rating

As a validity check of the actor’s facial emotional expression during the video clips, undergraduate students (N = 34) were asked to identify the actor’s emotion from a choice of emotions (Fear, Anger, Frustration, Sadness, Pain) and to rate the emotional intensity on a 5-point Likert-scale (with 1 being very low and 5 very high). All 34 students rated the actor as expressing sadness, with a mean intensity of 3.21 (SD = .69, range = 2–5).

#### Emotional perspective-taking: Puppet story task

A modified version of Wellman and Woolley’s (1990) puppet story task was utilized to assess children’s ability to reason about others’ emotions. Children were tested with six stories in which a child character was looking for a lost item. There were two stories for each of three possible outcomes: Finds-Wanted, in which the characters find what they were looking for; Finds-Nothing, in which the characters find nothing; and Finds-Substitute, in which the characters find something other than the object which they were looking for. The experimenter asked “Is ____ happy or is s/he sad?” and then encouraged the child to affix the character a face that showed how he or she felt. The child’s answer was coded as correct if s/he chose the sad face for the two Finds-Nothing and two Finds-Substitute stories, and the ‘happy’ face for the two Finds-Wanted stories.

#### Visual Perspective-Taking Task Level 1a (VPT Level 1a)

To be consistent with prior investigations [33, 40–41], two tasks were used to assess Level 1 visual perspective-taking, both of which were originally used by Flavell and colleagues (1981). In the first visual perspective-taking task, the child was shown a card on which a dog was pictured on one side and a cat on the other. The experimenter asked the child to name the animal on one side of the card, then turned the card over and asked the child to name the animal on the other side, praising and repeating the child’s correct responses. The experimenter then held the card perpendicular to the table, so that one side was visible to the child and the other side to the experimenter, and asked “Now, what animal can you see?” followed by “What animal can I see on my side?”. The child was not given any praise for either answer. The experimenter then flipped the card over and asked the same questions for the other animal (i.e., cat or dog). While the first questions about identifying animals served as familiarization trials, the answers to the last two questions were coded as correct if the child named the animal that the experimenter could see, resulting in a score that ranged from 0 to 2.

#### Visual Perspective-Taking Task Level 1b (VPT Level 1b)

In a second task, the child was shown a picture of a cartoon turtle flat on a table. Once the child identified the animal and its parts (feet and shell), the experimenter then took a blank card and held it perpendicular to the picture of the turtle, splitting the turtle so that the feet were only visible to the child, and that the shell was only visible to the experimenter. The child was then asked “What part of the turtle can I see?” and the card was then rotated so that the opposite parts were visible to the child and experimenter. The child was asked the same question about the next part (feet). After successfully identifying the turtle’s shell and feet, children’s answers to both questions were coded as correct (1 point) if they responded with correct part of the turtle visible to the experimenter, and incorrect (0 point) if they did not. As in the VPT Level 1a, scores ranged from 0 to 2.

## Results

With respect to the theory of mind tasks, the two visual Perspective-Taking tasks (Level 1a and Level 1b) were correlated (r(62) = .34, p < .01) and therefore scores were combined to increase variability (named “Visual Perspective-Taking Combined”–VPT Combined). The VPT Combined variable, the sum of the child’s scores on both VPT tasks (for a maximum of 4) was used in the subsequent regression analyses. See Table 1 for descriptive statistics and zero-order correlations. There was a positive correlation between emotional perspective-taking (M = 3.52, SD = 1.26) and the difference in looking time at the face (M = .16, SD = .17), r(49) = .38, p < .01, between visual perspective-taking (M = 1.75, SD = 1.40) and gender, r(62) = .37, p < .01 as well as between visual perspective-taking and looking at the eyes (M = -.02, SD = .19), r(49) = .30, p = .04 (Table 1). Next, examining correlations between differences in looking times on the eye-tracking task, we found a positive correlation between the difference in looking time at the face (M = .16, SD = .17), and the eyes (M = -.02, SD = .19), r(49) = .30, p = .04. However, looking at the eyes (M = -.02, SD = .19) negatively correlated with looking at the mouth (M = .21, SD = .21), r(49) = -.64, p < .01, as well as the distractors (M = -.15, SD = .25), r(49) = -.31, p < .05.

**Table 1:** Zero-order correlations among scores.

	Gender	EPT	VPT	Gender/EPT	Gender/VPT	Difference in Looking Time: Face	Difference in Looking Time: Eyes	Difference in Looking Time: Mouth	Difference in Looking Time: Distractors
Gender	-								
EPT	-.124	-							
VPT	.006	-.150	-						
Gender/EPT	-.048	.277*	-.019	-					
Gender/VPT	-.009	-.017	.230	-.142	-				
Difference in Looking Time: Face	.220	.382**	.054	.122	-.004	-			
Difference in Looking Time: Eyes	.396**	.208	.283*	.075	-.193	.493**	-		
Difference in Looking Time: Mouth	-.214	-.015	-.266	.048	.267	.182	-.638**	-	
Difference in Looking Time: Distractors	-.359**	-.154	.090	-.242	.141	-.301*	-.310*	.078	-
Mean	-	3.52	1.75	-.08	.00	3128.75	172.06	3040.80	-1200.39
Standard deviation	-	1.26	1.40	.65	.72	2023.07	2572.14	2546.36	1586.56

Note. N = 51. EPT = Emotion Perspective-Taking, VPT = Visual Perspective-Taking.

*p < .05.

**p < .01

**Fig 2:**
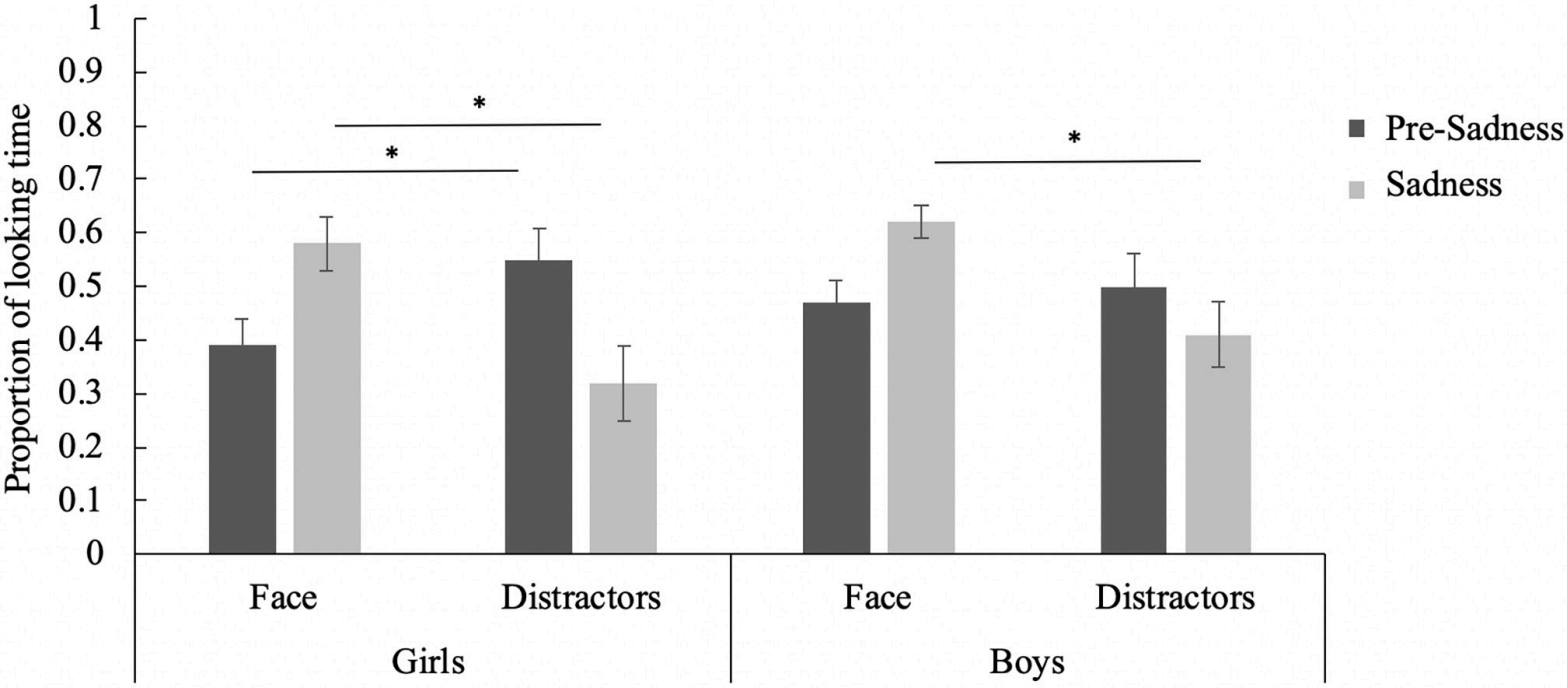
Proportion of looking time to the face and distractors during the pre-sadness and sadness segments for boys and girls. * p < .05.

Next, independent samples t-tests revealed no significant gender difference on the ToM tasks, both for the emotional perspective-taking, t(62) = .99, p = .33, d = .25, and for the visual perspective-taking, t(62) = .35, p .73, d = .09. A 2 (AOI) x 2 (Video Segment) X 2 (Gender) mixed analysis of variance was conducted to compare proportions of visual fixation time to the face and distractors (toys and hand) before and during the display of distress, with the between-subjects variable of gender and with age as a co-variate. An interaction between AOI and Segment was observed, F (1,48) = 4.30, p = .04 η^2^ = .08. Although non-significant, children spent a greater proportion of time looking at the distractors than the face before the ball was stolen (distractors: M = .52, SD = .32, face: M = .43, SD = .21, t(50) = -1.81, p = .08, d = .34, but a greater proportion of time looking at the face than the distractors after the ball was stolen (distractors: M = .36, SD = .31, face: M = .60, SD = .18 t(50) = 5.06, p < .01, d = .91). A 3-way interaction also emerged, F (1, 48) = 4.27, p = .04, η^2^ = .08. During the pre-sadness segment, girls looked at the distractors (M = .55, SD = .30) more than the face (M = .39, SD = .23, t(19) = -2.18, p = .04, d = .61), however no differences emerged for boys (face: M = .47, SD = .30, distractors: M = .50, SD = .33, t(30) = -.57, p = .58, d = .11). During the sadness segment, both boys and girls looked at the face more than the distractors (boys [face: M = .62, SD = .16, distractors: M = .41, SD = .34, t(30) = 3.42, p < .01, d = .77] girls [face: M = .58, SD = .21, distractors: M = .32, SD = .25, t(19) = 3.82, p < .01, d = 1.13]) (Fig 2).

**Fig 3:**
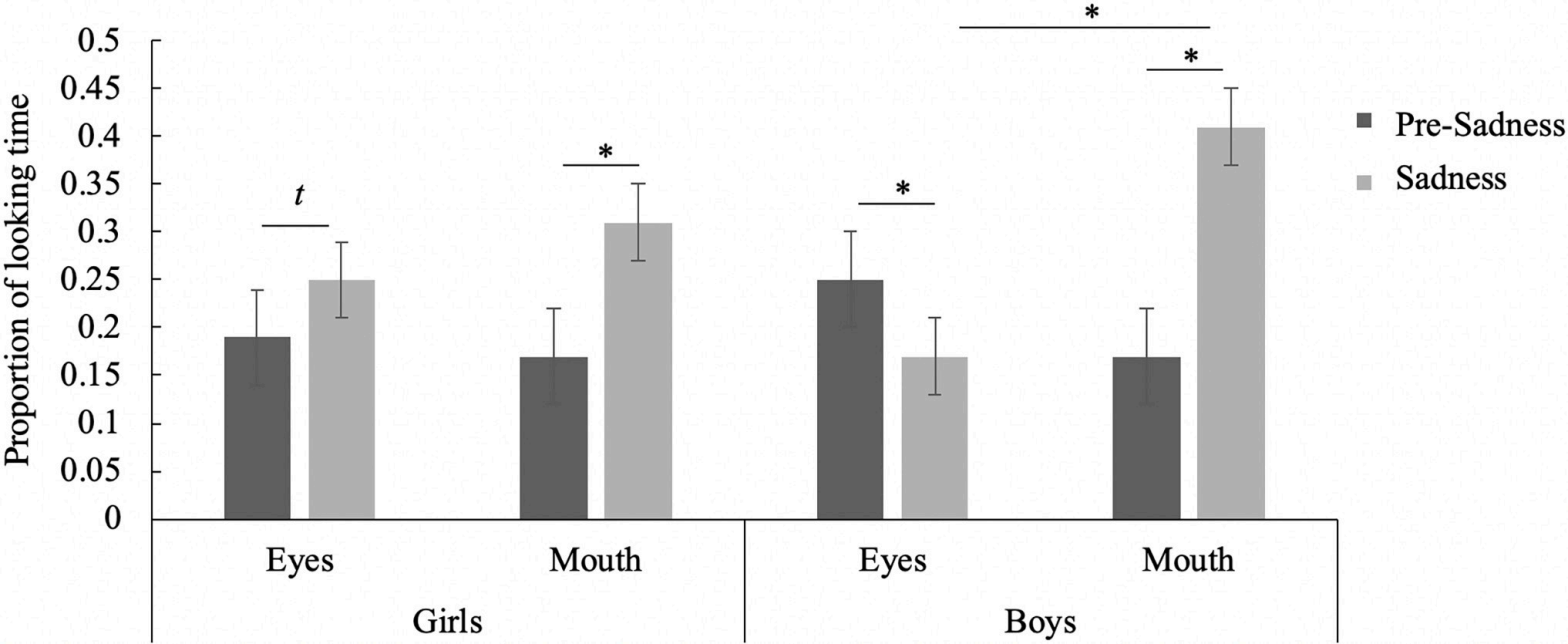
Proportion of looking time to the eyes and mouth during the pre-sadness and sadness segments for boys and girls. * p < .05.

We also examined changes in visual attention to internal features of the face (eyes and mouth) before and during the emotional changes across gender, and found significant 3-way interaction effects, (F (1,48) = 7.26, p < .01, η^2^ = .13). During the pre-sadness segment, both boys and girls gazed equally at the actor’s eyes and mouth (boys [eyes: M = .25, SD = .22, mouth: M = .17, SD = .15, t(30) = 1.49, p = .15, d = .42]; girls [eyes: M = .19, SD = .21, mouth: M = .17, SD = .15 t(19) = .31, p = .76, d = .11]). During the sadness segment, girls gazed equally at the eyes (M = .25, SD = .22) and the mouth (M = .31, SD = .24, t(19) = -.60, p = .55, d = .24), whereas boys looked longer at the mouth (M = .42, SD = .23) than the eyes (M = .17, SD = .20, t(30) = -3.66, p < .01, d = 1.16) (Fig 3). Further, while boys demonstrated a significant decrease in the proportion of looking time to the eyes from the pre-sadness segment (M = .25, SD = .22) to the sadness segment (M = .17, SD = .20), t(30) = 2.18, p = .04, d = .35, girls did not significantly change scanning of the eyes from the pre-sadness (M = .19, SD = .21) to the sadness segment (M = .25, SD = .22), t(19) = -1.86, p = .08, d = .32.

### Theory of mind in relation to eye gaze

Four hierarchical multiple regression analyses were conducted to assess the extent to which children’s theory of mind abilities predicted changes in their scanning patterns of the face expressing sadness. Gender was entered in Step 1, the two theory of mind variables were added in Step 2, and the interactions between gender and visual perspective-taking and between gender and emotional perspective-taking were added in Step 3. The outcome variables included the differences in proportion of looking times between the sadness segment as compared to the pre-sadness segments for face, eyes, mouth, and distractors (Tables 2, 3, 4 and 5, respectively).

**Table 2:** Summary of hierarchical regression analysis for variables predicting looking time to the face.

	Model 1			Model 2			Model 3		
Variable	B	SE B	ß	B	SE B	ß	B	SE B	ß
Constant	1871.58	848.49*		-764.25	1192.81		-780.32	1254.87	
Gender	903.04	571.75	0.22	958.30	534.89	0.23	956.16	546.55	0.23
EPT				663.69	221.53	0.39**	666.27	238.35	0.39**
VPT				122.66	185.55	0.09	126.43	190.97	0.09
Gender*EPT							-2.21	455.91	-0.001
Gender*VPT							-72.62	382.16	-0.03

Note. EPT = Emotion Perspective-Taking, VPT = Visual Perspective-Taking.

*p < .305.

**p < .401

Adj, R5 = .12 (p = .06)

**Table 3:** Summary of hierarchical regression analysis for variables predicting looking time to the eyes.

	Model 1			Model 2			Model 3		
Variable	B	SE B	ß	B	SE B	ß	B	SE B	ß
Constant	-2702.28	1009.60*		-5668.55	1417.57**		-5880.50	1435.89**	
Gender	2064.66	684.34	0.40**	2198.11	635.69	0.42**	2173.83	625.39	0.42**
EPT				498.07	263.27	0.23	534.87	272.74	0.25
VPT				579.46	220.51	0.32*	623.60	218.5	0.35**
Gender*EPT							-71.07	521.68	-0.02
Gender*VPT							-827.17	437.29	-0.23

Note. EPT = Emotion Perspective-Taking, VPT = Visual Perspective-Taking.

*p < .605.

**p < .701

Adj, R8 = .29 (p < .001)

**Table 4:** Summary of hierarchical regression analysis for variables predicting looking time to the mouth.

	Model 1			Model 2			Model 3		
Variable	B	SE B	ß	B	SE B	ß	B	SE B	ß
Constant	4580.48	1063.11**		5852.85	1574.38**		6300.63	1566.69**	
Gender	-1105.97	720.61		-1204.82	706.01	-0.23	-1173.10	682.36	-0.23
EPT				-68.34	292.39	-0.03	-160.43	297.58	-0.08
VPT				-506.34	244.90	-0.28*	-570.53	238.42	-0.32*
Gender*EPT							368.25	569.20	0.09
Gender*VPT							1070.57	477.12	0.30*

Note. EPT = Emotion Perspective-Taking, VPT = Visual Perspective-Taking.

*p < .905.

**p < .1001

Adj, R11 = .13 (p = .04).

**Table 5:** Summary of hierarchical regression analysis for variables predicting looking time to the distractors.

	Model 1			Model 2			Model 3		
Variable	B	SE B	ß	B	SE B	ß	B	SE B	ß
Constant	408.92	632.85		1033.96	962.31		745.67	976.36	
Gender	-1155.99	428.97	-0.36**	-1153.57	431.53	-0.36**	-1144.09	425.25	-0.36**
EPT				-211.59	178.72	-0.16	-131.74	185.45	-0.01
VPT				67.07	149.69	0.06	66.81	148.58	0.06
Gender*EPT							-549.36	354.72	-0.22
Gender*VPT							279.97	297.34	0.13

Note. EPT = Emotion Perspective-Taking, VPT = Visual Perspective-Taking.

*p < .05.

**p < .01

Adj, R^2^ = .13 (p = .04).

#### Looking time at face

The regression model predicting the difference in looking time at the actor’s face from before to after the ball was stolen approached significance, F (5, 45) = 2.33, p = .06, adj. R^2^ = .117. At step 2, emotional perspective-taking was a significant predictor. At step 3, the addition of the interaction terms did not explain significantly more variance. Therefore, children who were better at identifying a character’s emotional desires directed more visual attention to the actor’s face in the sadness segment relative to the pre-sadness segment (β = .39, t = 2.80, p < .01: see Table 2).

#### Looking time at eyes

The regression predicting difference in looking time at the actor’s eyes was significant, F (5, 45) = 4.99, p < .01, adj. R^2^ = .347. At step 1, gender was a significant predictor, and at step 2, VPT was significant and EPT was at trend level. The addition of both interaction predictors did not account for significantly more variance. Therefore, girls looked at the actor’s eyes more than boys (β = .42, t(49) = 3.48, p < .01) and children who had a better understanding of a character’s visual perspective and to a lesser extent, emotional desires, looked more at the actor’s eyes during the sadness segment, compared to the pre-sadness segment (visual perspective-taking: β = .35, t(49) = 2.85 , p < .01, emotional perspective-taking: β = .25, t(49) = 1.96, p = .06: Table 3).

#### Looking time at the mouth

The regression predicting difference in looking at actor’s mouth was significant F (5, 45) = 2.52, p = .04, adj. R^2^ = .132. At step 2, there was a significant effect of VPT, and at step 3, this was moderated by a significant gender X VPT interaction (β = .298, t(49) = 2.24, p = .03). Examined separately by gender, higher VPT combined score predicted less looking at the actor’s mouth for boys (β = -.49, t(49) = - 3.05 p < .01), but not for girls (β = .06, t(49) = .24, p = .81) (Table 4).

#### Looking time at the distractors

The final regression predicting difference in looking times at the distractors was significant, F (5, 45) = 2.51, p = .04, adj. R^2^ = .131. The only significant predictor was gender (β = -.356, t (49) = -2.69 p = .01). Boys directed more visual attention to the distractors than did girls (Table 5).

## Discussion

Research on the scanning patterns of adults when viewing emotional faces has revealed that adults tend to focus on the most informative areas of the face based on the emotion expressed [9–12]. While only a few studies examined these bottom-up mechanisms in young children, more extensive research examined how young children perceive emotional faces across emotional expressions. This research demonstrated that infants can discriminate between emotional expressions before their first birthday [4–5], and that young girls appear to be more attuned [38–39] and advanced at understanding the emotions of others when compared to boys [37]. In addition to bottom-up mechanisms in the scanning of emotional faces, many lines of evidence converge to indicate that top-down influences on perception, be it cognitive, social or emotional, should be considered a fundamental framework that supports visual perception [42–43]. Recently, some rudimentary form of expectation-based feedback, that is, the expectation of a sensory input propagating information to and receiving feedback from higher level processes has been reported in the occipital cortex of 6-month-old infants [44]. However, this top-down modulation has yet to be examined in young children’s scanning of visual faces, where it can be hypothesized that toddler’s emotional and visual perspective-taking abilities (emerging socio-cognitive skills) predict their scanning of a sad facial expression.

The current study therefore examined whether toddlers focus their attention on the face of a person within the context of a scene in which she displays a sad facial expression after a toy is taken away from her. More specifically, we examined whether increased visual attention to the eyes, the area of the face used by adults to recognize sadness [10], would be observed when children are exposed to a sad face, and if this differed based on their gender. Finally, we examined whether increased attention to the face as well as mouth and eyes could be predicted by children’s performance in tasks that require insight into the emotional or visual perspective of others.

As predicted, children as young as 32 months distributed their attention across all elements of the scene before the actor expressed sadness and then increased their visual fixations on the person’s face after she started showing a sad expression. Given that the actor gazed at the distractors during the pre-emotion segment, the increase of attention to the face may be a spurious artefact. Although this argument may explain the difference scores for looking at the face versus distractors, it cannot explain the different looking patterns for the eyes versus the mouth. Interestingly, the fact that girls spent more time looking at the distractors than boys during the pre-emotion segment would reflect girls’ increased attention to the face and gaze of the actor. This looking pattern suggests that very young children are adept at processing social information that is salient and necessary in order to infer the emotional states of others. However, there was a marked difference in the scanning of the face areas according to the gender of the child. Relative to boys, girls allocated more of their attention to the actor’s eyes, less to the actor’s mouth, and somewhat less to the other objects in the scene during the sadness segment as compared to the pre-sadness segment of the video. This finding is of importance, as the part of the actor’s face that changed most dramatically from the pre-sadness segment to the sadness segment was the mouth, as the corners turned from up (smiling) to down (sadness) while the lower lip protruded. Although this obvious configurational change appears to draw the attention of boys and girls to the mouth, as indicated by their looking times at the mouth during the sadness segment, it was the eyes, which are the most informative part of sad facial expressions [10–12], that kept girls’ attention. This is consistent with research documenting that, across the first two decades of life, females are more observant of facial expressiveness than males [38]. Further, this finding highlights differences between how boys and girls scan a sad facial expression. To our knowledge, this is the first study to document scanning of an emotional facial expression (i.e., sadness) following a negative event that included such young children. Our findings also extend previous work by showing that very young girls direct their attention towards the face area thought to be most informative about a person’s affective state, namely the eyes [45]. One could argue that these gender differences may be partly due to the fact that the agent in the video was female. However, while some researchers have shown no evidence of own-sex bias in face recognition in children [46], others have demonstrated that both male and female infants show preference for female body shape [47] and faces [48]. Adults also show no evidence of own-sex bias, demonstrating the same attentional bias toward male over female faces, especially in threat or anger contexts [49]. Further, at least two meta-analyses on the effect of gender on emotion processing have reported no such own-gender bias across a wide age range [50–51]. Nonetheless, replication of the current findings using a video of a male actor expressing the emotion would be desirable.

Another central goal of the current study was to examine whether variations in shifts in visual attention to the face and other aspects of the scene could be predicted by individual variability in theory of mind abilities. Specifically, we examined if toddlers’ looking towards the face, eyes, mouth and distractors present in the scene, was predicted by their emotional and visual perspective-taking skills. The strongest predictor for the toddlers’ looking pattern towards the actor’s face, as opposed to other elements of the scene, was their ability to predict the emotional reactions of a character who fulfills (happiness) or fails to fulfill (sadness) a goal. Our findings reveal that both girls and boys with more advanced emotional perspective-taking skills increased their focus on the actor’s eyes during the sadness segment, suggesting that they were attending to the most affectively informative component of the actor’s facial expression. This both validates Wellman and Wooley’s (1990) procedure, and points toward an early-emerging connection between toddlers’ understanding of others’ emotions and their scanning of emotional faces. Yet, it is noteworthy that non-emotional aspects of early theory of mind also appear to differently support attentiveness to emotional cues for boys and girls. That is, our findings reveal a main effect of better visual perspective- taking on looking toward the eyes for both girls and boys, but also less looking to the mouth for boys only. Having these visual perspective-taking skills, in addition to emotional perspective-taking skills, contribute to boys’ scanning of a sad facial expression, might help narrow the gap in emotional understanding between boys and girls [37]. This suggests that boys could be applying additional social-cognitive skills to guide their scanning of emotional faces. Interestingly, previous data was interpreted to suggest that boys may be using different processes to experience empathy, when compared to girls [52–53]. For example, Hinnant and O’Brien (2007) examined how emotional and cognitive control and cognitive and affective perspective-taking relate to 5-year-olds’ empathetic responses. They found that for 5-year-old boys, but not girls, empathy was related to cognitive control.

Although we did not examine toddlers’ empathetic responses in the current study, our findings converge with those of Hinnant and O’Brien (2007), as both studies highlight gender differences in the mechanisms that may be supporting the scanning of emotional facial expressions, and empathetic response in young children. It is plausible that the current results regarding the cognitive mechanisms supporting the scanning of emotional expressions in 2-year-old boys and girls are the precursors to those leading to a comparable empathetic response in boys and girls at age 5 [52, 54–55]. This conclusion is indirectly supported by findings demonstrating that the appropriateness of empathic responses improves over the second and third year of life [56], which could suggest that toddlers become more accurate in perceiving an actor’s affective cues and consequent needs. Future studies should examine how toddlers and older children scan empathy-inducing scenes and how their scanning patterns predict empathic responses. These studies are required in order to clarify the relation between emotional face processing and empathy-related responses such as sympathy and personal distress in boys and girls. Finally, as expected, the change in attention towards the distractors present in the scene (i.e., toy rings and frog) was not predicted by performance on either theory mind tasks. That is, young children’s emerging social-cognitive abilities do not direct their attention toward stimuli that are meaningless to understand the emotional states of others. Overall, these results offer further evidence for the specific association between early theory of mind skills and visual attention to emotional information, in addition to suggesting that boys and girls may be relying on a different combination of perspective-taking skills during their scanning of emotional facial expressions.

Taken together, the present findings suggest that toddlers’ attention to facial features is guided, as is the case for adults, by both bottom-up as well as top-down cognitive mechanisms. A shift of attention to the face when emotional expressions are displayed, particularly facial features that show significant changes (e.g. mouth) and features that are most informative based on the expressed emotion (e.g. eyes for sadness), reflects the bottom-up aspect of emotion processing. Given that the context was a negative event, toddlers’ looking patterns were predicted by their emotional perspective-taking skills (looking at the face) as well as by their visual perspective-taking skills (looking at the eyes). However, the current findings also demonstrate that there are gender differences in scanning emotional faces at this age. Girls looked more toward the actors' eyes whereas boys looked more toward the distractors and mouth, unless boys had better visual perspective-taking skills, which was associated with boys focusing less on the mouth. Future research should, in children as well as in adults, examine if different parts of the face are more or less informative at different points in the expression formation. A more refined analysis of dynamic emotion processing would allow for a better understanding of emotional facial expression processing. Finally, as this was a non-experimental study conducted at a single time, we cannot rule out other factors that may have contributed to the observed relations between visual perspective-taking skills and visual attention to facial expression of emotion. To examine the causal and directional relation between the development of theory of mind and the scanning of emotional faces, we suggest that future studies examine this relation with experimental and repeated-measures longitudinal designs.

## Supporting information

S1 Dataset (XLSX)
